# Seismic waveform tomography with shot-encoding using a restarted L-BFGS algorithm

**DOI:** 10.1038/s41598-017-09294-y

**Published:** 2017-08-17

**Authors:** Ying Rao, Yanghua Wang

**Affiliations:** 1 State Key Laboratory of Petroleum Resources & Prospecting, China University of Petroleum (Beijing), Beijing, 102249 China; 2 Centre for Reservoir Geophysics, Department of Earth Science and Engineering, Imperial College London, London, SW7 2BP UK

## Abstract

In seismic waveform tomography, or full-waveform inversion (FWI), one effective strategy used to reduce the computational cost is shot-encoding, which encodes all shots randomly and sums them into one super shot to significantly reduce the number of wavefield simulations in the inversion. However, this process will induce instability in the iterative inversion regardless of whether it uses a robust limited-memory BFGS (L-BFGS) algorithm. The restarted L-BFGS algorithm proposed here is both stable and efficient. This breakthrough ensures, for the first time, the applicability of advanced FWI methods to three-dimensional seismic field data. In a standard L-BFGS algorithm, if the shot-encoding remains unchanged, it will generate a crosstalk effect between different shots. This crosstalk effect can only be suppressed by employing sufficient randomness in the shot-encoding. Therefore, the implementation of the L-BFGS algorithm is restarted at every segment. Each segment consists of a number of iterations; the first few iterations use an invariant encoding, while the remainder use random re-coding. This restarted L-BFGS algorithm balances the computational efficiency of shot-encoding, the convergence stability of the L-BFGS algorithm, and the inversion quality characteristic of random encoding in FWI.

## Introduction

Full-waveform inversion (FWI) is used to extract information from seismic waveforms to reconstruct a subsurface model of the Earth with complex velocities. The inverted velocity images often demonstrate high resolution and high accuracy^1, 2^. However, their computational cost obstructs the wide application of FWI, especially for the inversion of seismic reflection data, which are routinely acquired during exploration geophysics^3^. In FWI methods, the subsurface model is updated iteratively following the update direction, which is defined in terms of the gradient. This gradient vector is the first-order derivative of the FWI objective function with respect to the unknown model parameters, and is computed for each shot separately and is formed by cross-correlation between the incident wavefield and the adjoint wavefield. These two wavefields are simulated for each seismic shot individually^4^. Thus, this process requires a very high operational speed, particularly for practical three-dimensional (3D) seismic data with thousands of shots.

The shot-encoding technique assigns different weights, either +1 or −1, to all the shots randomly and sums them into a super shot, greatly reducing the number of wavefield simulations needed for FWI^5–9^. While this shot-encoding strategy improves the efficiency of FWI, it undesirably enhances crosstalk noise, which is caused by the interference among the individual shots within the super shot.

For FWI, the most commonly used inversion methods include the gradient-based method and the quasi-Newton method. The latter involves the Hessian matrix, which is the second-order derivative of the FWI objective function. The limited-memory BFGS (L-BFGS) algorithm is one example of a quasi-Newton method^10, 11^, where BFGS refers to the Broyden-Fletcher-Goldfarb-Shanno algorithm for updating the Hessian matrix or its inverse, and limited-memory means this algorithm does not store these matrices explicitly. The L-BFGS algorithm has proven to be more effective than the gradient-based method^12–14^. However, if the shot-encoding strategy is adopted for FWI, the standard L-BFGS algorithm will cause an unstable convergence^15, 16^. This effect occurs because the calculation in each iteration requires the difference in the gradient between consecutive iterations. These gradients are inconsistent since each iteration uses different shot-encoding values and in turn different objective functions. The restarted L-BFGS algorithm developed in this paper ensures the stability of the inversion and the imaging quality of the shot-encoded FWI. Furthermore, a higher efficiency and fewer memory requirements ensure the applicability of the shot-encoded FWI method to 3D seismic data.

## Shot-encoded FWI

During the application of FWI, the model is updated along the negative direction of the gradient vector^17^. Using the adjoint-state method^4, 18^, the gradient is obtained through a cross-correlation between the wavefield from forward modelling and the wavefield from residual back-propagation. Back-propagation uses exactly the same computing engine as employed by forward modelling. Therefore, if there are *N*_*S*_ shots in the seismic field data, then there are 2 × *N*_*S*_ wavefield simulations that must be executed at each iteration.

In seismology, a shot gather refers to a group of seismic traces recorded by a series of receivers for which the recorded wavefield has originated from a single source. To reduce the number of wavefield simulations in the inversion process, the shot gathers are multiplied by either +1 or −1 randomly and are then stacked into a super-shot gather^5^. Equivalently, the source signatures are multiplied by random codes and are subsequently excited simultaneously to simulate forward wave propagation. For the calculation of the gradient, the residual of the waveform between the shot-encoded synthetic wavefield and the shot-encoded observed wavefield is backward propagated and cross-correlated with the forward wavefield. This cross-correlation will exhibit a crosstalk effect between different shots, which can directly deteriorate the quality of FWI imagery^7^.

For typical shot-encoded FWI, seismic shots are assigned into different super shots^16, 19, 20^. The shots contained within a super shot should be randomly encoded rather than simply superimposed so that the crosstalk effect can be offset by the randomness of encoding. The objective function for the shot-encoded FWI is defined in terms of the weighted wavefields according to the weights in the encoding procedure. Then, the gradient of the objective function consists of both the ordinary gradient (i.e., without shot-encoding) and an extra term representing the crosstalk between different shots. Fortunately, this crosstalk term also clearly indicates that an increase in the randomness of the shot-encoding can effectively suppress the crosstalk effect. This quality improvement occurs because the weights *w*_*i*_ used in the encoding procedure are randomly generated values of +1 and −1, and the sum of a large number of *w*_*i*_ *w*_*j*_ (i.e., the multiplication between different encoded shots) in the crosstalk term has an expectation of zero^8, 9^.

The inversion algorithm used in shot-encoded FWI must be stochastic. Because the model update procedure is stochastic, both gradient-based and Newton-type methods used in conventional deterministic inversions must be properly adjusted to fit within a shot-encoded framework. The restarted L-BFGS algorithm proposed in this paper exploits the features of shot-encoding and the L-BFGS algorithm and uses these features to enhance the computational efficiency and image quality of FWI by mitigating the crosstalk effect.

## Restarted L-BFGS algorithm

In FWI, the subsurface model is updated iteratively, and thus, the theoretical wavefield that is calculated based on the subsurface model gradually matches the field observations. Hence, the objective function is commonly described as

1 $$\varphi ({\bf{m}})=\frac{1}{2}{\Vert {{\bf{d}}}_{{\rm{obs}}}-{{\bf{d}}}_{{\rm{cal}}}({\bf{m}})\Vert }_{2}^{2},$$

where **d**_obs_ is the vector of the observed seismic data, and **d**_cal_(**m**) is the vector of the synthetic data calculated based on the model, **m**. This objective function is minimised gradually through iterative model updates. The model update is defined by the gradient according to a precondition of the inverse Hessian matrix in the form of^21^

2 $${{\bf{m}}}_{k+1}={{\bf{m}}}_{k}-{\alpha }_{k}{{\bf{B}}}_{k}{{\bf{g}}}_{k},$$

where *k* is the iteration number, *α*_*k*_ is the step length, **B**_*k*_ is the inverse Hessian matrix, and **g**_*k*_ = ∇*ϕ*(**m**_*k*_) is the gradient vector. Since it exploits the inverse Hessian matrix, this is a quasi-Newton method.

In a BFGS algorithm, the inverse Hessian matrix **B**_*k*_ is derived recursively from **B**_*k*−1_ using the following relation^22^

3 $${{\bf{B}}}_{k}=({\bf{I}}-\frac{{{\bf{z}}}_{k-1}{{\bf{y}}}_{k-1}^{T}}{{{\bf{y}}}_{k-1}^{T}{{\bf{z}}}_{k-1}})\,{{\bf{B}}}_{k-1}({\bf{I}}-\frac{{{\bf{y}}}_{k-1}{{\bf{z}}}_{k-1}^{T}}{{{\bf{y}}}_{k-1}^{T}{{\bf{z}}}_{k-1}})+\frac{{{\bf{z}}}_{k-1}{{\bf{z}}}_{k-1}^{T}}{{{\bf{y}}}_{k-1}^{T}{{\bf{z}}}_{k-1}},$$

where **I** is the identity matrix, **y**_*k*−1_ is the difference in the gradient between two consecutive iterations, and **z**_*k*−1_ is the model difference. Because of its recursive nature, **B**_*k*−1_ does not need to be stored, and **B**_*k*_ can be calculated quickly and adaptively based on the gradient differences {**y**_*k*−1_, **y**_*k*−2_, **y**_*k*−3_, …} and the model differences {**z**_*k*−1_, **z**_*k*−2_, **z**_*k*−3_, …} from the previous iterations. Therefore, Equation (3) represents the limited-memory version of the BFGS algorithm (i.e., L-BFGS).

If ${{\bf{y}}}_{k-1}^{T}{{\bf{z}}}_{k-1} > 0$, the inverse Hessian matrix **B**_*k*_ is positive definite. This is because in the following formula

4 $${{\bf{v}}}^{T}{{\bf{B}}}_{k}{\bf{v}}={({\bf{v}}-\frac{{{\bf{z}}}_{k-1}^{T}{\bf{v}}}{{{\bf{z}}}_{k-1}^{T}{{\bf{y}}}_{k-1}}{{\bf{y}}}_{k-1})}^{T}{{\bf{B}}}_{k-1}({\bf{v}}-\frac{{{\bf{z}}}_{k-1}^{T}{\bf{v}}}{{{\bf{z}}}_{k-1}^{T}{{\bf{y}}}_{k-1}}{{\bf{y}}}_{k-1})+\frac{{({{\bf{z}}}_{k-1}^{T}{\bf{v}})}^{2}}{{{\bf{y}}}_{k-1}^{T}{{\bf{z}}}_{k-1}},$$

if **B**_*k*−1_ > 0, both terms on the right-hand side are nonnegative, while the first term is zero only if **v** = 0, and the second term is zero only if ${{\bf{z}}}_{k-1}^{T}{\bf{v}}=0$. This positive definite property ensures that the update −**B**_*k*_ **g**_*k*_ (Equation 2) is in a descent direction, and consequently the model update during the inversion will converge. If shots are re-coded at each iteration, two gradients **g**_*k*_(**m**_*k*_, **w**_*k*_) and **g**_*k*−1_(**m**_*k*−1_, **w**_*k*−1_) are calculated from the objective functions with different sets of encoding, which are **w**_*k*−1_ and **w**_*k*_. For the gradient difference

5 $${{\bf{y}}}_{k-1}={{\bf{g}}}_{k}-{{\bf{g}}}_{k-1},$$

even if the step length *α*_*k*−1_ is sufficiently small, the condition ${{\bf{y}}}_{k-1}^{T}{{\bf{z}}}_{k-1} > 0$ is difficult to satisfy. Therefore, shot-encoding will induce instability and potentially non-convergence during FWI.

**Figure 1:**
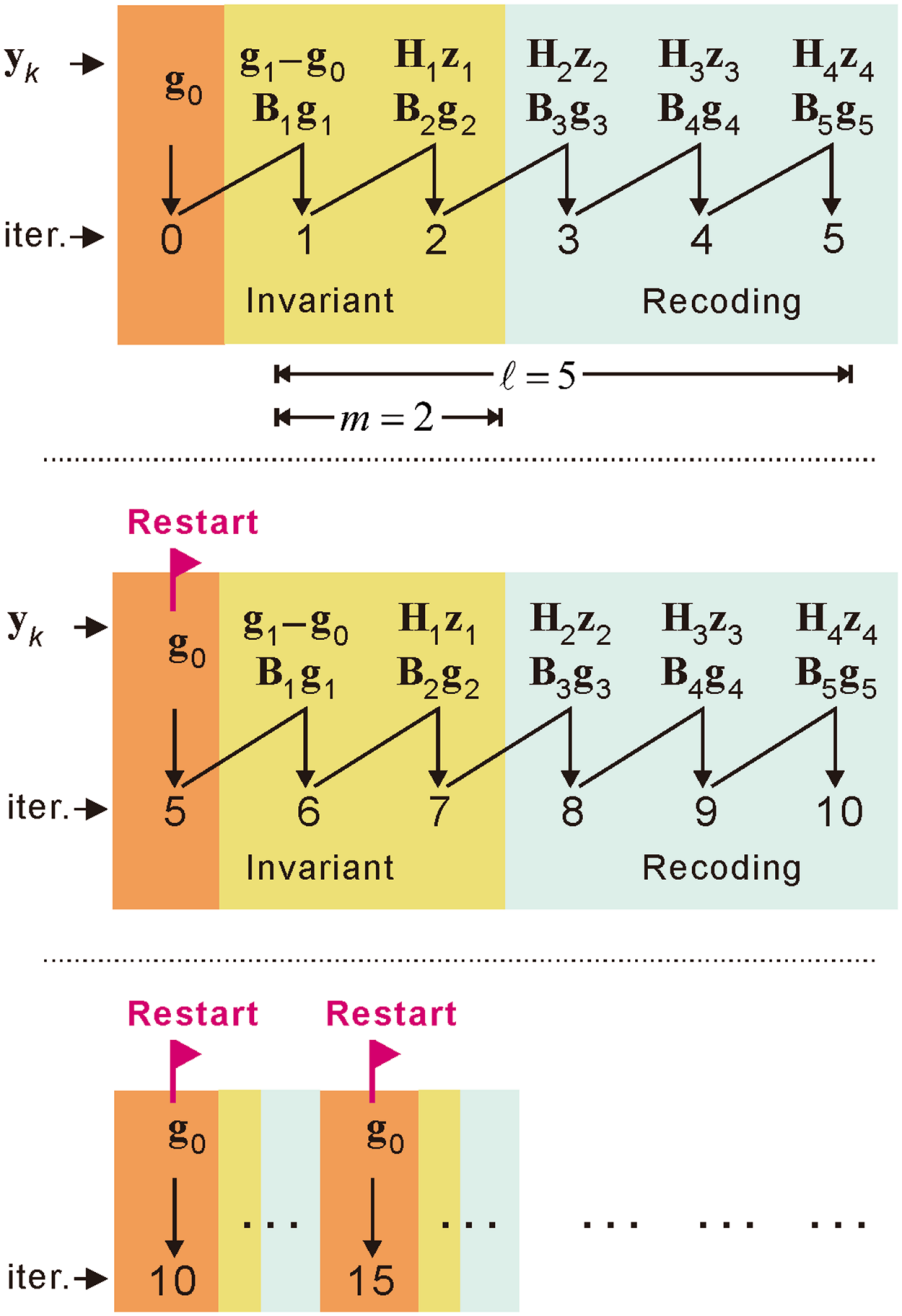
The restarted L-BFGS algorithm. The implementation of the L-BFGS algorithm is restarted after a number of iterations ($\ell$). Within the segment between two starts, only the coding of the first few iterations (*m*) is kept unchanged (yellow) and is the same as the previous one (orange). The shots for the rest of the iterations (grey) are re-coded with a sufficient randomness to suppress crosstalk effects. Within a restarted segment, the gradient differential is given by the secant equation **y**_*k*−1_ = **H**_*k*−1_ **z**_*k*−1_ except for the first iteration, where **y**_0_ = **g**_1_ − **g**_0_. Both **H**_*k*−1_ **z**_*k*−1_ and **B**_*k*_ **g**_*k*_ are calculated using the limited-memory formulas.

Here, we propose the restarted L-BFGS algorithm (Fig. 1) containing the following three distinguishing features.

1. Structurally, the L-BFGS calculation is restarted after every $\ell$ iterations. The total number of L-BFGS iterations is divided into a series of segments, and each segment consists of $\ell$ iterations. The recursive L-BFGS calculation is only based on the gradient differences **y**_*k*_ and model differences **z**_*k*_ within the individual segment. Therefore, the segment interval $\ell$ is also referred to as the storage length in the L-BFGS algorithm (i.e., for storing the vectors **y**_*k*_ and **z**_*k*_).
2. Within a segment, the shot-encoding of the first few iterations is kept invariant (yellow) and is the same as the previous iteration (orange). A new reassignment of the weights (either +1 or −1) is triggered for the remainder of the iterations (grey). We denote *m* as the number of iterations that employ invariant coding. Generally, $2\le m < \ell$, which maximises the overall randomness of shot-encoding to effectively suppress the crosstalk effect. Meanwhile, maintaining the weights of coding as invariant for a few of iterations, rather than recoding at every iteration, will significantly improve the convergence speed and stability during the inversion.
3. For the first iteration, the gradient difference is calculated by **y**_0_ = **g**_1_ − **g**_0_. For the rest of the iterations, the gradient difference is calculated based on the following secant equation:

6 $${{\bf{y}}}_{k-1}={{\bf{H}}}_{k-1}{{\bf{z}}}_{k-1},$$

where **H**_*k*−1 _= ∇^2^ *ϕ*(**m**_*k*−1_) is the Hessian matrix in the *k*−1*-*th iteration. Equation (6) represents the gradient differential rather than the gradient difference. Using the gradient differential will improve the accuracy and, in turn, the stability of convergence of the FWI.

Note that the secant equation in Equation (6) differs slightly from the conventional secant equation used in the BFGS algorithm. The latter imposes a condition^23^ wherein **H**_*k*_ is close to the current matrix **H**_*k*−1_ and presents the following approximation as the secant equation:

7 $${{\bf{y}}}_{k-1}={{\bf{H}}}_{k}{{\bf{z}}}_{k-1}.$$

This approximation can be justified because

8 $${{\bf{y}}}_{k-1}\equiv {{\bf{g}}}_{k}-{{\bf{g}}}_{k-1}=\frac{{{\bf{g}}}_{k}-{{\bf{g}}}_{k-1}}{{{\bf{m}}}_{k}-{{\bf{m}}}_{k-1}}({{\bf{m}}}_{k}-{{\bf{m}}}_{k-1}),$$

wherein the fraction can be treated as either a forward difference or a backward difference, i.e.,

9 $$\frac{{{\bf{g}}}_{k}-{{\bf{g}}}_{k-1}}{{{\bf{m}}}_{k}-{{\bf{m}}}_{k-1}}\approx {{\bf{H}}}_{k}\approx {{\bf{H}}}_{k-1}.$$

However, the approximated secant equation in Equation (7) cannot be used to directly calculate **y**_*k*−1_, as it depends on **H**_*k*_ and not **H**_*k*−1_. In this paper, we use secant Equation (6) to calculate **y**_*k*−1_.

To calculate the Hessian matrix, we use the DFP (Davidon-Fletcher-Powell) method^23^, which represents the Hessian matrix based on the gradient difference and the model difference as follows:

10 $${{\bf{H}}}_{k}=({\bf{I}}-\frac{{{\bf{y}}}_{k-1}{{\bf{z}}}_{k-1}^{T}}{{{\bf{y}}}_{k-1}^{T}{{\bf{z}}}_{k-1}})\,{{\bf{H}}}_{k-1}({\bf{I}}-\frac{{{\bf{z}}}_{k-1}{{\bf{y}}}_{k-1}^{T}}{{{\bf{y}}}_{k-1}^{T}{{\bf{z}}}_{k-1}})+\frac{{{\bf{y}}}_{k-1}{{\bf{y}}}_{k-1}^{T}}{{{\bf{y}}}_{k-1}^{T}{{\bf{z}}}_{k-1}}.$$

Exploiting its recursive characteristic, we derive the following formula:

11 $$\begin{array}{rcl}{{\bf{H}}}_{k} & = & {{\bf{V}}}_{k-1}^{T}{{\bf{V}}}_{k-2}^{T}\cdots {{\bf{V}}}_{1}^{T}{{\bf{H}}}_{0}{{\bf{V}}}_{1}\cdots {{\bf{V}}}_{k-2}{{\bf{V}}}_{k-1}\\  &  & +{\rho }_{1}{{\bf{V}}}_{k-1}^{T}{{\bf{V}}}_{k-2}^{T}\cdots {{\bf{V}}}_{2}^{T}{{\bf{y}}}_{1}{{\bf{y}}}_{1}^{T}{{\bf{V}}}_{2}\cdots {{\bf{V}}}_{k-2}{{\bf{V}}}_{k-1}\\  &  & \vdots \\  &  & +{\rho }_{k-2}{{\bf{V}}}_{k-1}^{T}{{\bf{y}}}_{k-2}{{\bf{y}}}_{k-2}^{T}{{\bf{V}}}_{k-1}\\  &  & +{\rho }_{k-1}{{\bf{y}}}_{k-1}{{\bf{y}}}_{k-1}^{T},\end{array}$$

where ${\rho }_{k}=1/{{\bf{y}}}_{k}^{T}{{\bf{z}}}_{k}$ and ${{\bf{V}}}_{k}=({\bf{I}}-{\rho }_{k}{{\bf{z}}}_{k}{{\bf{y}}}_{k}^{T})$. According to the secant equation, where **H**_*k*_ **z**_*k*_ = **y**_*k*_, we define the initial Hessian matrix **H**_0_ as

12 $${{\bf{H}}}_{0}={{\bf{y}}}_{0}{{\bf{y}}}_{0}^{T}{[{{\bf{z}}}_{0}{{\bf{y}}}_{0}^{T}]}^{-1}\approx \frac{{{\bf{y}}}_{0}^{T}{{\bf{y}}}_{0}}{{{\bf{z}}}_{0}^{T}{{\bf{y}}}_{0}}{\bf{I}}.$$

Equation (11) does not need to store matrices and is a limited-memory version of the DFP formula. This equation is similar to the L-BFGS formula, which uses the same recursive form as Equation (11) for the inverse Hessian matrices **B**_*k*_, for *k* = 0, 1, …, and does not need to store these matrices as well. Therefore, in shot-encoded FWI, we use the L-BFGS and DFP algorithms and calculate **B**_*k*_ **g**_*k*_ and **H**_*k*_ **z**_*k*_ based only on the vectors **y**_*k*_ and **z**_*k*_, which are stored within the segment of $\ell$ iterations.

## Results

### Accuracy of the gradient differential

The difference in the gradients is an approximation to the gradient differential. The numerical errors contained within **y**_*k*−1_ include any strong perturbations in gradients **g**_*k*_ and **g**_*k*−1_. However, the secant equation is an accurate solution of the differential of the gradient functional. The gradient differential based on **H**_*k*−1_ **z**_*k*−1_ is more stable than the subtraction of any two gradients. The accuracy of **H**_*k*−1_ **z**_*k*−1_, which replaces **g**_*k*_ − **g**_*k*−1_, ensures the steady convergence of the iterative inversion.

Gradient differentials are used as constraints for the calculation of the Hessian matrix **H**_*k*_ and its inverse **B**_*k*_, which both have a similar limited-memory recursive formula. Because of their recursive characteristics, the influence of the initial ${{\bf{y}}}_{k-\ell }$ within the invariant encoding procedure gradually decreases in the subsequent iterations wherein the shots are encoded randomly. Therefore, the L-BFGS calculation must be restarted after a given number of iterations.

### Randomness vs. crosstalk

**Figure 2:**
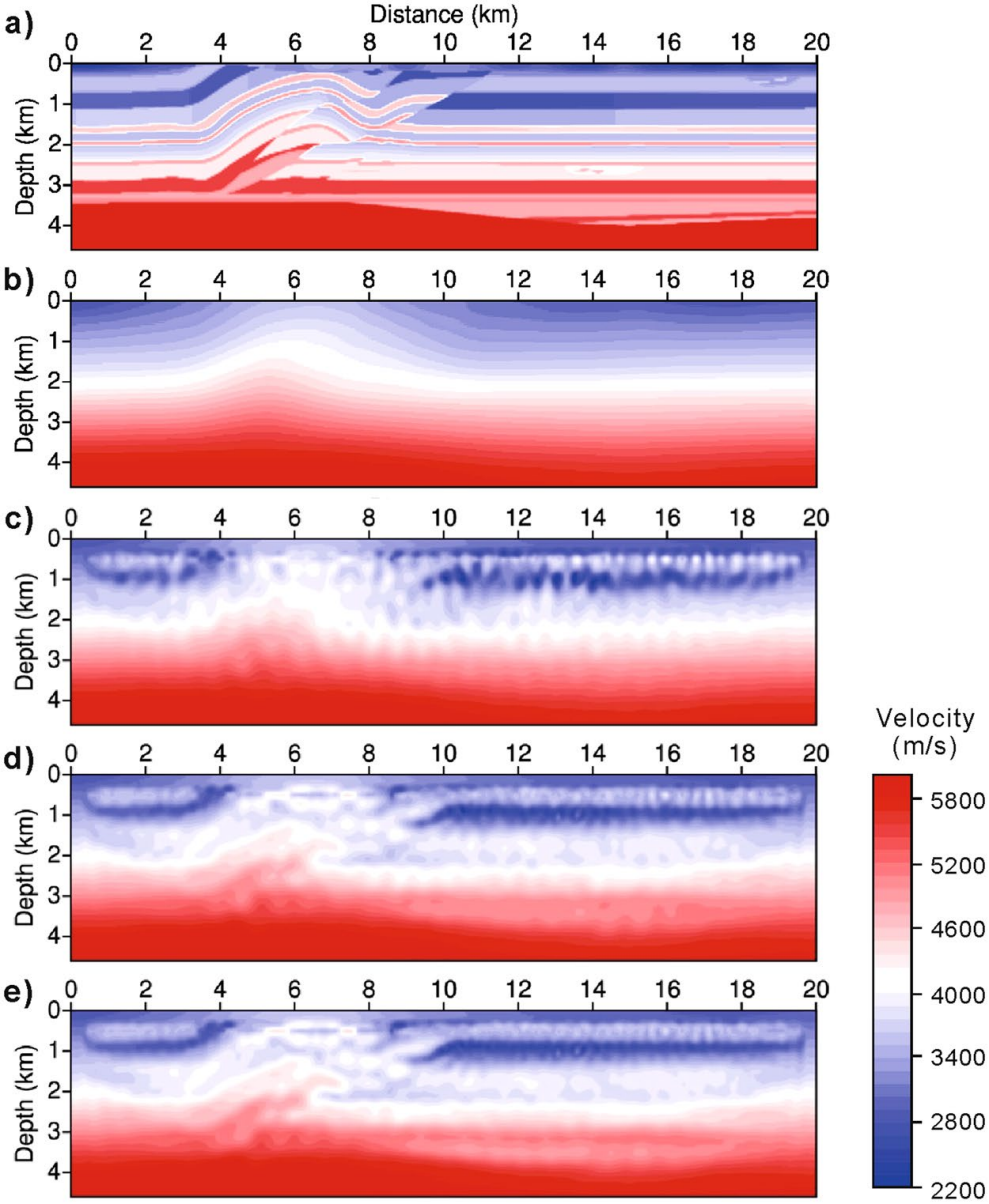
Randomness vs. crosstalk. (**a**) An overthrust model. (**b**) The initial model for FWI. (**c**,**d**,**e**) The inversion results at the 10^th^, 50^th^ and 100^th^ iterations, using the data set of the lowest frequency band (2−4 Hz). Crosstalk noise is gradually suppressed along with the increased randomness in the restarted L-BFGS algorithm.

Only randomness can suppress the crosstalk effect in shot-encoded FWI. A 2D line of an SEG/EAGE overthrust model^24^ (Fig. 2a) is used to test the effectiveness of the restarted L-BFGS algorithm. The model is partitioned into 401 × 93 cells in the horizontal and vertical directions with a 50-m cell size. There are 191 shot points laid atop the model. The spatial interval between the shots is 100 m. The source signature is a Ricker wavelet^25^ with a dominant frequency of 7 Hz. Each shot gather consists of 401 receivers. For shot-encoded FWI, all 191 shots are grouped into a single super shot.

The multi-scale inversion method is adopted^26, 27^, for which the data have been divided into four frequency bands: 2−4, 4−6, 6−8 and 8−10 Hz. Therefore, the FWI procedure consists of four stages. The initial model for the first inversion of the data set within the lowest frequency band is the smoothed version of the true model (Fig. 2b). The initial model for the inversion of any data set within a higher frequency band is the inversion result from the lower frequency band. The inversion of each frequency-band data set is executed over 200 iterations.

Figure 2 also displays the results at the 10th, 50th and 100th iterations, using the data set from the lowest frequency band. These results clearly demonstrate the presence of the crosstalk effect within shot-encoded FWI. The shallow horizontally layered structure appears to be truncated by noise into multiple short segments. The deeper horizontal interface appears curved with numerous fluctuations. However, this crosstalk effect decreases with an increase in the number of iterations as a consequence of an increase in the accumulated randomness of encoding.

**Figure 3:**
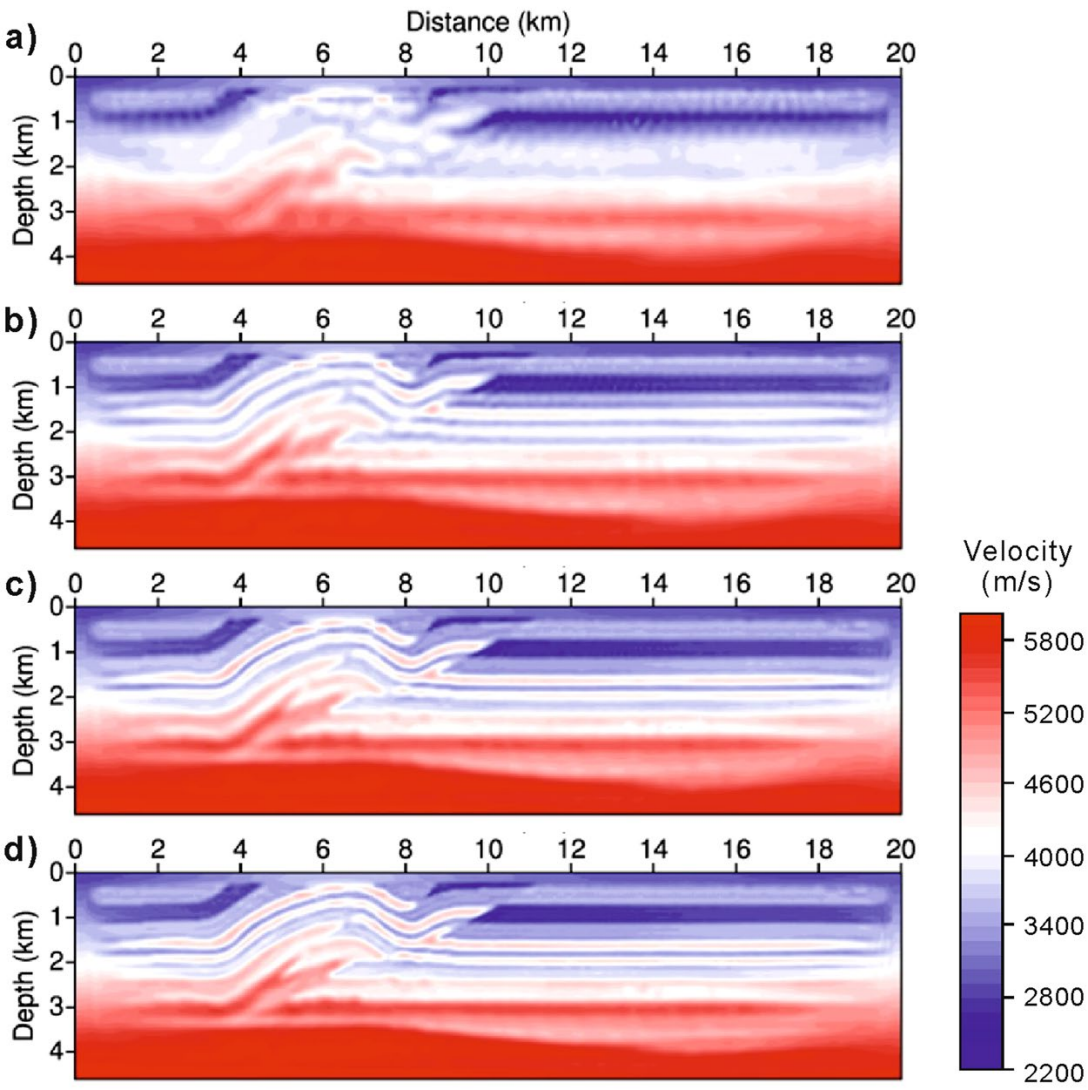
Shot-encoded FWI with the restarted L-BFGS algorithm. (**a**,**b**,**c**,**d**) Reconstructed velocity profiles corresponding to the respective inversion stages of four frequency-band data sets (2−4, 4−6, 6−8, 8−10 Hz). The crosstalk effect is eventually suppressed because of the cumulative randomness in the shot-encoding.

The crosstalk effect will be effectively suppressed in the final inversion results because the randomness of shot-encoding is further increased stage by stage along with an increased number of L-BFGS iterations during the multi-scale inversion. In the multi-scale inversion shown in Fig. 3, a total of 800 iterations have been executed. In the final result of the four-stage inversion, the fault structure and shallow layers have been successfully reconstructed using shot-encoded FWI.

**Figure 4:**
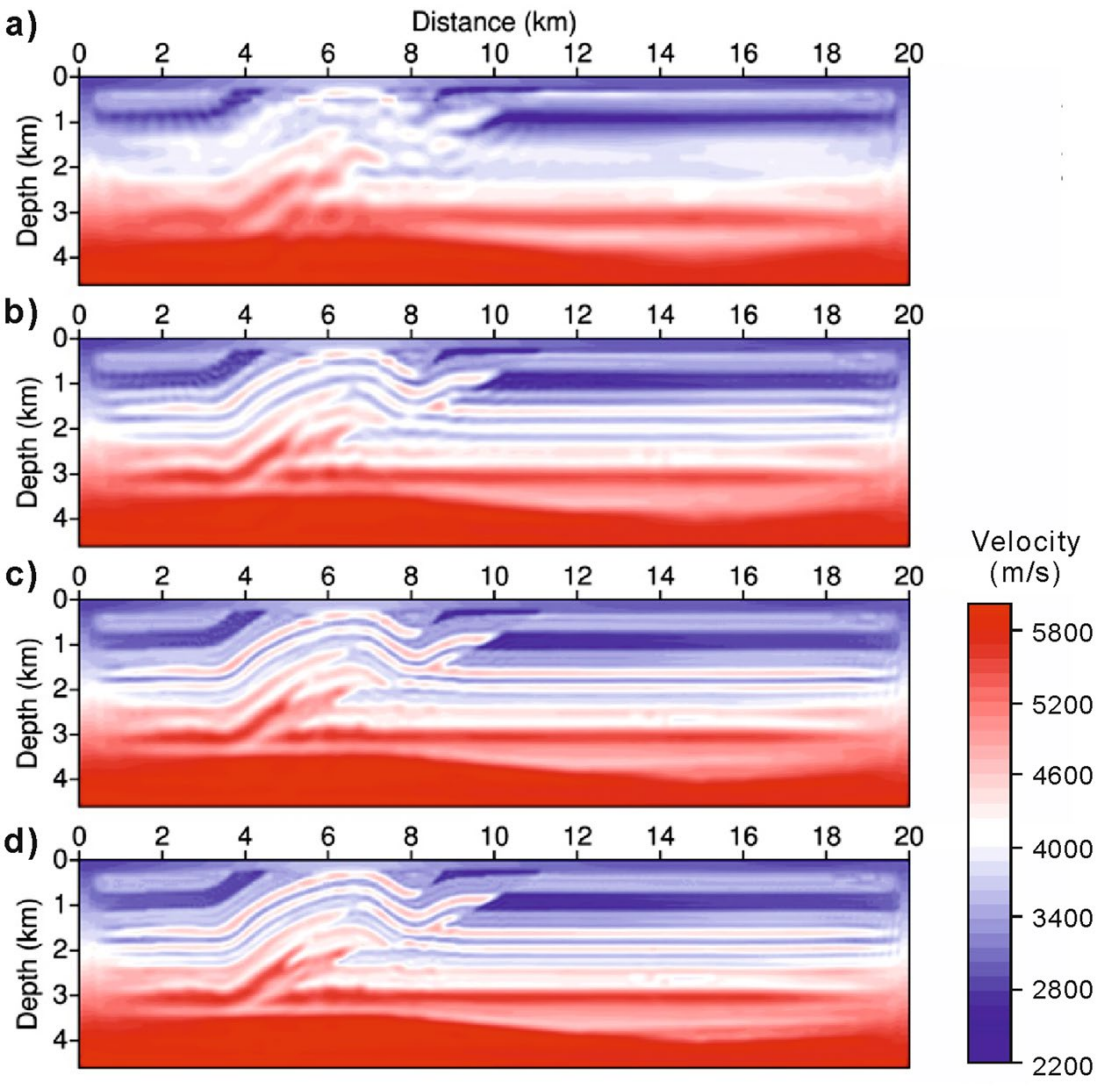
Velocity models reconstructed by FWI without shot-encoding. **(a,b,c,d)** Results corresponding to the respective data sets of four frequency bands. These results are the benchmarks used for the shot-encoded FWI with the restarted L-BFGS algorithm proposed in this paper.

For comparison, Fig. 4 displays the results of standard FWI, without shot-encoding, using the standard L-BFGS algorithm. For each data set, the inversion is executed over 30 iterations. Therefore, a total of 120 iterations are executed. The main structures are clearly recovered in the FWI model. These results are used as a benchmark for verifying the effectiveness of shot-encoded FWI.

**Figure 5:**
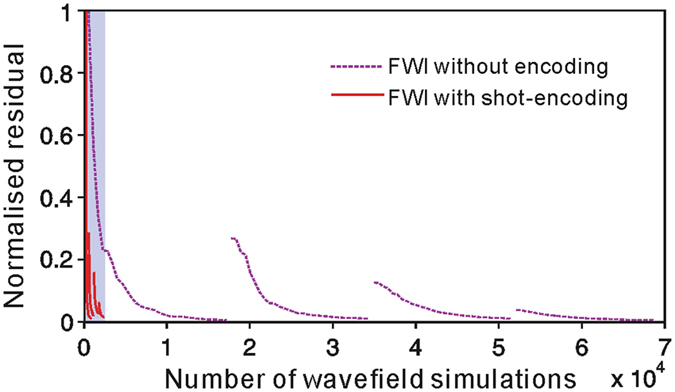
Number of wavefield simulations. Convergence rates of FWI without shot-encoding (purple dashed lines) and of shot-encoded FWI using the restarted L-BFGS algorithm (red solid lines within the shadowed zone). The horizontal axis denotes the number of wavefield simulations, and the vertical axis is the normalised residual. Each line segment corresponds to an inversion of a data set for a particular frequency band.

Shot-encoded FWI utilising the restarted L-BFGS algorithm (Fig. 3) can produce results similar to those of FWI without shot-encoding utilising the standard L-BFGS algorithm (Fig. 4). The linear features stretching from the top to the bottom in the final results confirm that both the restarted L-BFGS algorithm and the standard L-BFGS algorithm possess the same reconstruction ability during waveform inversion. However, the efficiency of these two inversions differs significantly. The computational efficiency of FWI is determined by the actual number of wavefield simulations required. These wavefield simulations include wave back-propagation and step-length calculations for every iteration. Figure 5 shows the number of wavefield simulations required to achieve convergence. The total number of simulations in the restarted L-BFGS algorithm (<0.25 × 10^4^, shaded region in Fig. 5) is substantially less than that in the standard L-BFGS algorithm (7 × 10^4^). Therefore, the restarted L-BFGS algorithm fully reflects an accelerated shot-encoding inversion procedure. Compared with FWI without shot-encoding, the restarted L-BFGS algorithm significantly reduces computational costs.

### Applicability to 3D FWI

As the restarted L-BFGS algorithm can improve image quality by suppressing the crosstalk effect and can simultaneously reduce computational costs and storage requirements, shot-encoded FWI is shown to be applicable to cases involving 3D data sets.

The size of the actual 3D overthrust SEG/EAGE model is 4.65 × 20 × 20 km^3^. The model is partitioned into 93 × 401 × 401 grids with a cell size of 50 × 50 × 50 m^3^. The source signature is a Ricker wavelet with a domain frequency of 7 Hz. There are 400 shots spread out over the surface at an interval of 1 × 1 km^2^. Each shot gather consists of 32,761 traces. In the inversion, every 100 shots are assembled into a super shot.

**Figure 6:**
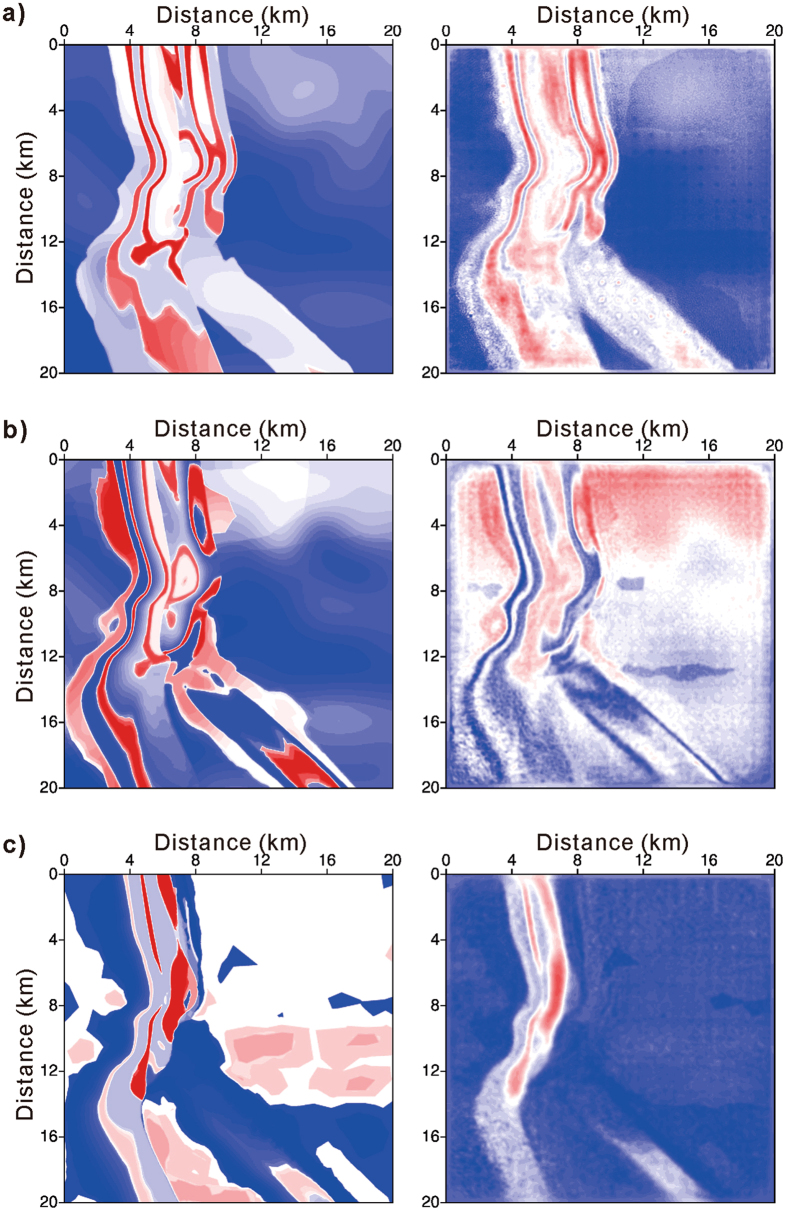
Shot-encoded FWI applicability to a 3D model. (**a**) Velocity slices at a depth of 1 km. (**b**) Velocity slices at a depth of 1.5 km. (**c**) Velocity slices at a depth of 2 km. The left column is the true velocity model, and the right column is the velocity model reconstructed using shot-encoded FWI.

The multi-scale inversion method is adopted for the shot-encoded FWI of the four frequency bands similar to that in the 2D example. The initial model is a Gaussian smoothed version of the true velocity model. For the inversion of each frequency band, 100 iterations are executed, and the restarted L-BFGS algorithm is used with $\ell =5$ and *m* = 2. Figure 6 displays the resulting velocity slices at different depths to compare the true velocity model and the reconstructed velocity model. At depths of 1.0 and 1.5 km, the morphology of an ancient channel is clearly reconstructed. At a depth of 2 km, the main body of the channel can also be inverted, although there is limited wave coverage.

## Conclusions

A restarted L-BFGS algorithm for shot-encoded FWI is proposed to improve the efficiency, enhance the stability and reduce the memory requirement for seismic waveform tomography.

In a standard L-BFGS algorithm, the recursive calculations require the difference of the gradients between consecutive iterations. If shot-encoding is conducted differently in different iterations, the gradients will be inconsistent, and the inversion will not converge steadily. The restarted L-BFGS algorithm replaces the difference in the gradients with an accurate gradient differential using the secant equation. Its accuracy leads to steady convergence. The algorithm also enhances the flexibility of the restart mode of the L-BFGS algorithm with shot-encoding. Because of sufficient randomness in the shot-encoding in the restarted L-BFGS algorithm, crosstalk noise is eventually suppressed through the iteration of the implemented L-BFGS algorithm.

This breakthrough ensures that shot-encoded FWI will be applicable to the vast quantity of existing seismic reflection data that have been acquired by exploration geophysicists. It also holds enormous potential in teleseismic research by preceding teleseismic tomography based on travel time residuals, as there is a better chance to generate high-resolution crustal images using this method and local earthquakes, where the sources are found inside the model, though their exact location and mechanism must also be determined.
